# Investigation of the Influence of Washing on the Physical and Mechanical Properties of Polymer Materials for Bandages

**DOI:** 10.3390/polym17111552

**Published:** 2025-06-02

**Authors:** Maja Somogyi Škoc, Slavica Bogović, Antonija Čulina, Iva Rezić Meštrović

**Affiliations:** 1 Department of Materials, Fibres and Textile Testing, University of Zagreb Faculty of Textile Technology, 10000 Zagreb, Croatia; antonija.culina.4@gmail.com; 2 Department of Clothing Technology, University of Zagreb Faculty of Textile Technology, 10000 Zagreb, Croatia; slavica.bogovic@ttf.unizg.hr; 3 Department of Applied Chemistry, University of Zagreb Faculty of Textile Technology, 10000 Zagreb, Croatia; iva.rezic@ttf.unizg.hr

**Keywords:** polymer materials, medical textiles, compression bandages, compression garments, physical–mechanical properties, 3D body scanning, wash

## Abstract

An elastic bandage or tensor bandage is widely known as a flexible medical device made of polymer materials. It is usually made of cotton and reinforced with elastic yarns. Depending on the therapy and clinical picture, elastic bandages are used for compression treatment and as support bandages. The aim of this work was to carry out a washing process and investigate its influence on the physical–mechanical properties of elastic bandages. The washing process was carried out at 40 °C with 25, 50, 60, and 70 wash cycles using Autowash 311L. The elastic bandages were subjected to a tensile load. The tensile strength, elongation, elasticity, and structural properties of the elastic bandages were determined. The results of the elongation show that the values increase with the wash cycles (in standard soap) and decrease after 70 cycles (in standard soap without phosphate). The tensile strength values are highest after 25 wash cycles. The results of the cyclic elasticity measurements show that the elastic bandages do not lose their elongation properties after the first cycle. After the second to fifth cycle, the samples do not return to their original or almost original dimensions when the tensile load is stopped. In addition, an analysis of the data from the 3D leg scanning and an approximation of the compression of the bandages was performed. The circularity and compression values after 60 washes remain within the limits in which the compression bandage can fulfill the compression function.

## 1. Introduction

Medical textiles, as part of the broader category of technical textiles, are an extremely important field of research and an indispensable part of modern disease management and our lives [1]. The increasing number of elderly people, the rising prevalence of chronic diseases, the growing need for surgical interventions, and economic progress in industrialized and developing countries [2] primarily influence today’s accelerated development of medical textiles. In addition, developments in textile technology and medical procedures have led to the growth and development of medical products ranging from single-thread sutures to complex composite structures for bone replacement, tissue engineering, drug delivery systems, etc. [3]. Today, medical textiles include a variety of different textile structures designed and manufactured for use in a wide range of medical applications. Depending on the application, medical textiles are categorized into health and hygiene products, extracorporeal devices, implantable materials, non-implantable materials, intelligent textiles for medicine and care, textiles in the healthcare sector, and components for controlling environmental hygiene [2]. Non-implantable materials include wound dressings, plasters, pressure bandages, bandages, etc. [4]. An elastic bandage or tensor bandage is widely known as a flexible medical device made of fabric reinforced with elastic yarns [5]. It can be applied to different parts of the body with different anatomies and has a therapeutic effect. Depending on the therapy and clinical picture, elastic bandages are used for compression treatment and as support bandages.

Most of the reviewed papers investigated elastic bandages from a medical point of view [6,7,8,9], while a smaller number of papers investigated compression after 10 cycles of hand and machine washing [10], multiaxial elongation [11], dynamic hysteresis coefficient (DHC) and elongation [12], as well as elasticity and burst strength [13]. In addition, in one study, stockings were washed by hand and in the machine (10 cycles) and their compression was measured [14]. The results show that the cotton components significantly deteriorate after repeated washing. The compression on the target body segment is measured or approximated with a sensor [7,9,10,12,14,15]. In view of the fact that there is a great deal of research into the use, testing, and design of compression garments, it can be concluded that the manufacture of compression garments or parts thereof is attracting a great deal of attention from scientists in various disciplines. The large number of different polymer materials used in the manufacture of compression garments, the different compression that needs to be achieved on one part of the body, and the extremely different sizes and shapes of the human body pose challenges for the manufacture of functional compression garments. From the user’s point of view, the biggest problem is the selection of a garment size that fully corresponds to body measurements and shape and at the same time achieves the prescribed compression in order to benefit from the positive effects of compression therapy [7,13,14,16,17,18,19,20]. 

A compression bandage, on the other hand, is not subject to any restrictions in terms of clothing size and can be considered a more favorable option for use in compressing body parts. The main problem with the use of compression bandages is the subjective determination of compression by wrapping the bandage in one, two, or more layers, whereby the compression can vary. To ensure uniform compression, some manufacturers add graphic elements that form regular shapes (circles or squares) when the bandage is properly stretched [7,21,22].

In studies analyzing elastic bandages from a medical perspective, some authors investigated their influence on post-operative recovery [16] while other authors investigated them as a compression therapy for venous leg ulcers [17]. In one study, the authors evaluated the effects of using compression bandages or stockings compared to no compression on the healing of venous leg ulcers [18]. In another study, the authors analyzed the benefits of compression with elastic bandages compared to calf massage for the prevention of venous thromboembolism and provided a retrospective evaluation [19]. Recent studies show that the development of an injectable, highly adhesive hydrogel bandage with controllable oxygen production and anti-inflammatory functions is of great value in preventing the formation of chronic wounds caused by hypoxia and cold in high-altitude areas [20]. In general, it can be said that elastic bandages are chosen for their performance rather than their construction. Elastic bandages are woven or knitted and are designed to provide a prescribed level of compression defined by the classification of compression bandages [23].

Proper hygiene of elastic bandages is essential for safe use, as unclean bandages can cause the growth of bacteria and fungi, which can lead to dermatitis, irritation, or infection. Regular washing ensures hygienic cleanliness by reducing microorganisms and odors, but frequent washing can also affect the mechanical properties of the bandage.

From the above-mentioned literature and other reviewed papers available to the authors, it can be concluded that the topic dealt with in this article has not yet been researched. The aim of this work was, therefore, to carry out a different number of washing cycles for elastic bandages and then investigate the influence of washing on the physical–mechanical properties of the elastic bandages (by cyclic elasticity measurements and the determination of tensile strength and elongation). Repeated washing is very important. It is known that frequent washing cycles can cause a change in the fiber surface due to the swelling capacity of the fibers in the detergent bath, which is superimposed by mechanical movement. In our case, repeated washing can cause elastic bandages to lose their mechanical strength, cotton fibrils to loosen, rubber threads to deteriorate, elasticity to decrease, etc. The focus of this study was on determining the number of washes that an elastic bandage can withstand without significantly changing its physical–mechanical properties. It is expected that this work will provide significant new information and new elements in the current understanding of elastic bandages and their physical and mechanical properties after different washing cycles, which is important for the efficiency of the application. Another focus of this work was to determine the approximate values for the compression, i.e., to calculate them according to the adapted Laplace law. This is very important as the compression bandage is wrapped with a certain amount of overlap, resulting in at least two layers of bandage material and variable compression.

## 2. Materials and Methods

### 2.1. Preparation of the Elastic Bandages

Woven elastic bandages were used for compression treatment and as support bandages. The elastic bandage examined in this article can be used on any part of the body—including the legs, arms, and torso—that requires compression or support. It is typically applied to high-movement areas such as the knees and elbows, but can also be used to stabilize other joints and muscles.

The fiber composition and dimensions of the elastic bandage were determined. The mass per unit area was determined according to ISO 3801 [24]. The thickness was determined in accordance with ISO 5084 [25].

### 2.2. Preparation of Washing Procedure

**Figure 1:**
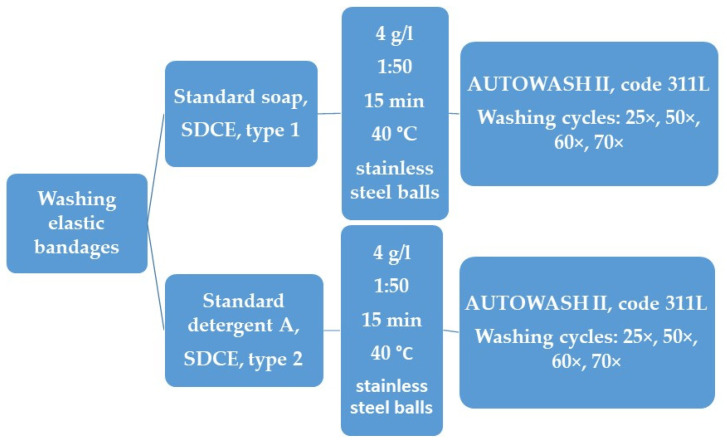
The experimental flowchart for the washing procedure.

Since the main objective of the research of this work was to determine the maximum number of washes that an elastic bandage can withstand (without significantly altering its physical–mechanical properties), a simulation was carried out by washing in AUTOWASH II code 311L (Mesdan S.p.A., Puegnago del Garda (Bs), Italy) and in accordance with ISO 6330:2021 [26]. The standard detergents were selected in accordance with the ISO 105-C10 standard [27]. Standard soap (SDCE type 1), phosphate-free ECE reference detergent A (SDCE type 2), and non-corrodible stainless steel balls (a washing test agitator material, approx. 6 mm in diameter) from SDC Enterprises Ltd. (Holmfirth, England) were used. The powdered standard soap was free of fluorescent brighteners. The phosphate-free ECE reference detergent A in powder form was also free of fluorescent brighteners. The standard soap was selected with the aim of simulating the type of washing carried out by most users (the elderly population)—washing in a solution of textile soap. For each wash, the same amount of soap or detergent was used, i.e., 4 g/L, with a mixing ratio of 1:50, at a temperature of 40 °C for 15 min (Figure 1). The temperature of 40 °C was chosen in accordance with the manufacturer’s instructions. The elastic bandages tested were subjected to a series of wash cycles of 25×, 50×, 60×, and 70× guided by the knowledge of how to control the quality of the washing process on the standard cotton fabric prescribed in DIN 53 919 so that the standard cotton fabric is washed 25 or 50 times, using the control method in [28,29]. The washing cycles (25×, 50×, 60×, and 70×) were determined empirically and based on the reviewed literature [30,31,32]. In addition, the sample was observed after each wash cycle. When a visually perceptible change and loosening occurred, the washing process was stopped (70×).

### 2.3. Determination of Tensile Properties

#### 2.3.1. Determination of Maximum Force and Elongation

A standardized test of the tensile strength and elongation of elastic bandages was carried out. The test of tensile strength and elongation was performed in accordance with ISO 13934-1:2008 [33]. The test was performed on a constant rate of extension strength testing machine, Tensolab 3000 (Mesdan S.p.A., Puegnago del Garda (Bs), Italy). Five specimens measuring 200 × 50 mm were prepared from each sample (unwashed sample, washed samples—25×, 50×, 60×, and 70×). The longer dimension corresponds to the direction of the length direction. The measured length of the test sample (distance between the clamps) was 100 mm. A load-controlled test was performed in which the load rate was controlled so that the load increase per unit time was constant (dP/dt = const). The speed of movement of the clamps was 100 ± 10 mm per minute. The test results are given in the form of the following values: breaking force [N] and elongation at break [%] with the calculation of statistical indicators. The three basic statistical indicators, arithmetic mean, standard deviation, and coefficient of variation, were calculated.

Student’s *t*-test (also called the T-test) was used to compare the means between two groups (unwashed and washed samples), with no multiple comparisons required as a unique *p*-value is observed. It was used to test whether the difference in mean values between the two groups was statistically significant. The null hypothesis states that both mean values are statistically equal, while the alternative hypothesis states that both mean values are not statistically equal, i.e., that they are statistically different from each other [34].

#### 2.3.2. Determination of the Elasticity of Fabrics

**Figure 2:**
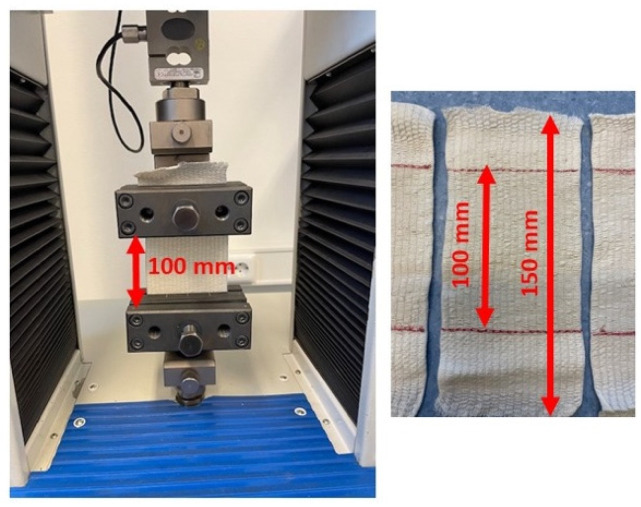
Appearance of the elastic bandage sample prepared for the test in the clamps of the dynamometer.

A standardized test of the elasticity of fabrics was carried out in accordance with Method A ISO 20932-3:2018 [35]. Method A is intended for a narrow test specimen of specific length dimensions, which is stretched at a constant speed and a specific force for a set number of cycles. Method A is used for the quality assurance of products. The test was also performed on a constant rate of extension strength testing machine, Tensolab 3000 (Mesdan S.p.A., Puegnago del Garda, Italy). The elastic bandages were cut to a length of 150 mm (Figure 2) and consisted of five test specimens.

The required limit values for the cycles between the measuring length and the corresponding load according to ISO 20932-3:2018 were determined. The load was determined using the mass per unit area (251 g m^−2^) and was 74.0 N. In the Tensolab 3000 software, an open module was selected for the hysteresis, with which the desired hysteresis cycles were set. This meets the requirements of international standards such as ISO 20932-3:2018. Five cycles were selected for each sample in accordance with the manual and the ISO mentioned; the load was 74.0 N and the recovery time was 1 min. The extension and retraction speed of the sample was set to 100%/min. The gauge length was (100 ± 1) mm. Each cycle was divided into 4 phases: the loading phase, the pause under load, the unloading phase, and the pause at the end of the unloading phase. The test results are given in the form of the following values: extension at maximum force (E [mm]), elongation (S [mm]), permanent deformation (C [%]), recovered elongation (D [%]), and elastic recovery (R [%]). The calculations were defined by ISO 20932-3:2018 [35]:

(1) $$S=\frac{E-L}{L}\cdot 100\;\left[\%\right]$$

where S is the elongation, E is the extension [mm] at maximum force in the last cycle (fifth cycle), and L is the initial length.

(2) $$C=\frac{Q-P}{P}\;\cdot 100\;\left[\%\right]$$

C is the permanent deformation, Q is the distance between the applied reference marks or, if a preload is used, the unrecovered elongation [mm] after a specified recovery time, and P is the initial distance between the applied reference marks or, if a preload is used, the initial length [mm]. The equation is as follows:

D = (100 − C) [%](3)

where D is the recovered elongation

(4) $$R=\frac{D}{S}\;\cdot 100\;\left[\%\right]$$

where R is the elastic recovery.

The results are also shown using typical cycle diagrams.

#### 2.3.3. D Scanning of the Human Body

**Figure 3:**
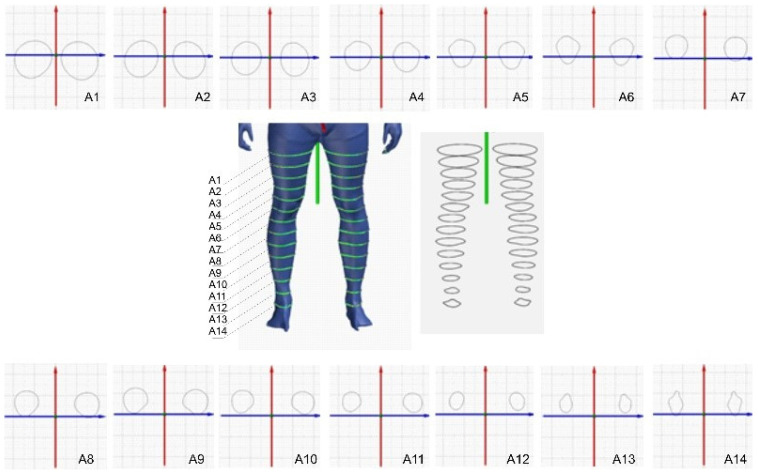
Three-dimensional scanning of the human body and the determination of body cross-sections.

A scan of the human body was generated with a Vitus Smart 3D scanner (Human Solutions GmbH, Kaiserslautern, Germany) to determine body shapes. The 3D scanning resulted in a point cloud with approximately 500,000 spatial coordinates, and the scanning area was 1000 × 800 × 2040 mm. The focus of this study is on the shapes of the cross-sections of the legs. Figure 3 shows a part of the point cloud related to the lower part of the body, where the shapes of the legs were analyzed based on the cross-section. A total of 14 planes were defined on which the cross-sections were extracted. The planes have a distance of 5 cm. The perimeters and areas of each cross-section were measured and the circularity of the shapes was calculated according to the following formula [36,37]:

(5) $$\mathrm{Cc}=\frac{4\Pi A}{P^{2}}$$

where Cc is the circularity, A is the area, and P is the perimeter.

The values obtained on the basis of expression (5) can be used to determine the extent to which the observed cross-section is compared to a circle. The circularity is in the range 0< Cc ≤ 1. If Cc = 1, it is a perfect circle. Lower values of Cc indicate shapes that deviate from a perfect circle in terms of either circumference or area. Once the circular shape was calculated, the compression of the bandage on the observed body part was calculated, taking into account the physical and mechanical properties of the compression bandage. The compression was calculated for each cross-section of the body. The modified Laplace’s law was used to calculate the compression as follows [15,21,38]:

(6) $$\mathrm{Pr}=\frac{Tn}{RW}$$

where Pr is the pressure (Pa), n is the layer of the bandage, W is the width of the bandage (m), T is the fabric tension (N), and R is the radius of the curvature of the limb (m).

### 2.4. Determination of the Morphology of the Materials

The surface properties of the samples were determined using the Dino-Lite microscopy system. The instrument used was the Dino-Lite Pro AM413T (Torrance, CA, USA) with extended software functions via the DinoCapture 2.0 software. The Dino-Lite Pro AM413T (Torrance, CA, USA) is a digital microscope with a resolution of 1.3 megapixels that can achieve a magnification of up to 200×. The morphological structure of the material, including the thickened and thinned areas of the yarns and fibers, can be observed.

## 3. Results

A sample of elastic bandages from the manufacturer with the largest market presence in the authors’ region was selected. For this work, the number of batches was reduced to one on the basis of the preliminary work, and the largest series from the year 2024 was selected [39]. The aim was to gain the best possible insight into the tested properties of the elastic bandages. The original dimensions of the elastic bandages used were 10 cm × 10 m. The composition showed that the elastic bandages consisted of 95% cotton fibers and 5% rubber (synthetic polyisoprene). The mass per unit area was determined to be 251 g m^−2^. The thickness was determined to be 1.34 mm. The elastic bandages were opened, unpacked, and suspended in a controlled atmosphere for 24 h immediately before the test. Table 1 contains the codes of the samples used in this work.

### 3.1. Results of the Construction Characteristics

The resistance of bandages primarily determines the behavior of polymers, i.e., of cotton fibers (cellulose) and rubber threads under the influence of various chemicals, but also of detergents, which is the subject of this work. The influence of the washing process on bandages must be considered via the composition of the raw material, the construction characteristics, and the combined effect of Sinner’s factors during washing (mechanics, chemicals, temperature, and processing time) [40,41,42]. This causes the cotton fibrils to loosen. The fibril release potential depends on the type of material (textile), the texture (loose or dense), the type of yarn, and the type of fibers.

The results of our study can be seen in the structure of the cotton fibers, in which areas of different order are present, i.e., amorphous and crystalline areas. Non-crystalline areas in cotton fibers play an important role as they are accessible to some external chemical compounds such as water molecules, detergents, dyes, etc., so that physical–chemical processes can take place in these areas [40]. It is precisely in these areas that initial changes in the mechanical deformation of the fibers first occur due to the action of forces. However, in products such as our elastic bandages, the density between the cotton threads decreases as the number of washes increases and the cotton fibers fibrillate, causing the elastic threads to fray and deteriorate.

**Figure 4:**
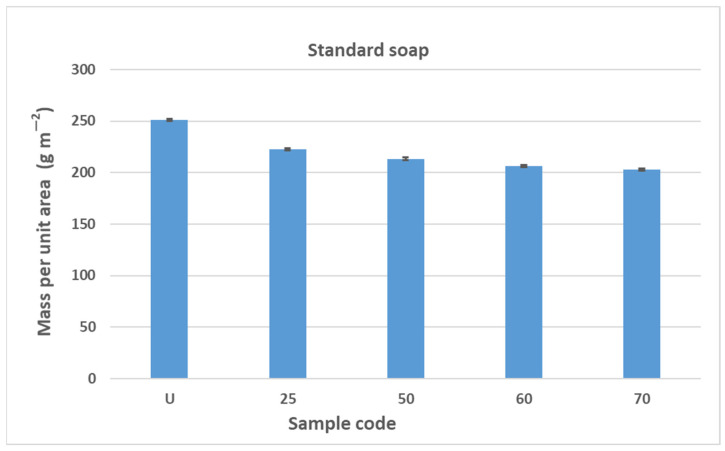
The results of the determination of the mass per unit area of the elastic bandage of the unwashed sample and after 25, 50, 60, and 70 washing cycles in standard soap.

**Figure 5:**
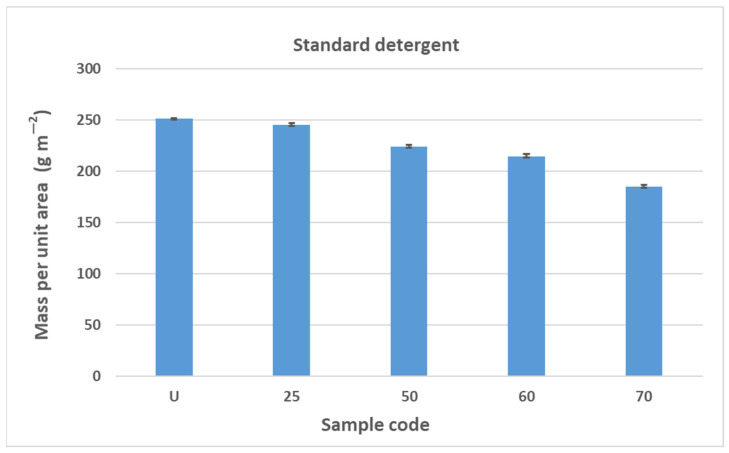
The results of the determination of the mass per unit area of the elastic bandage of the unwashed sample and after 25, 50, 60, and 70 washing cycles in standard detergent.

**Figure 6:**
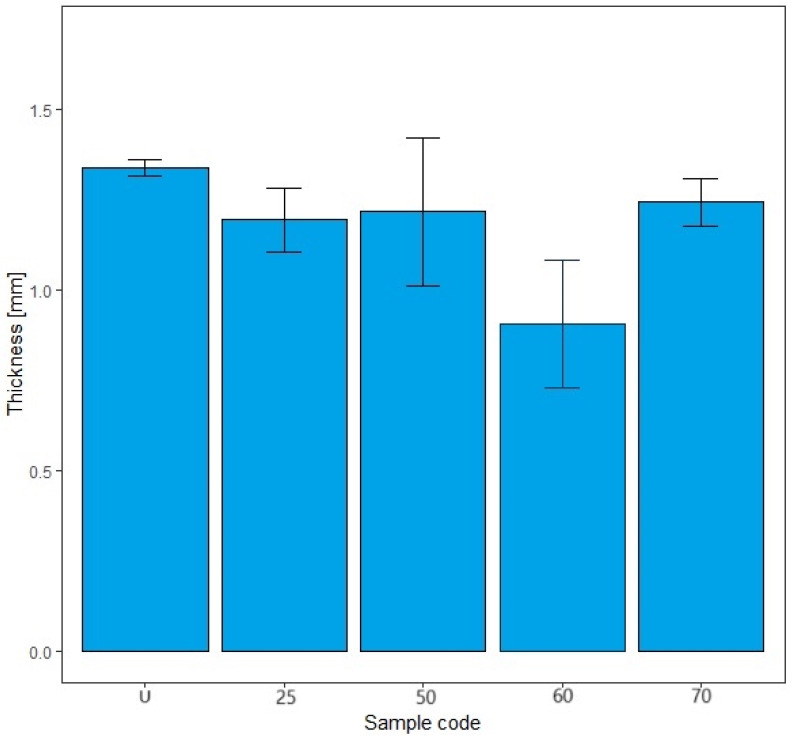
The results of the determination of the thickness of the elastic bandage of the unwashed sample and after 25, 50, 60, and 70 washing cycles in standard soap.

**Figure 7:**
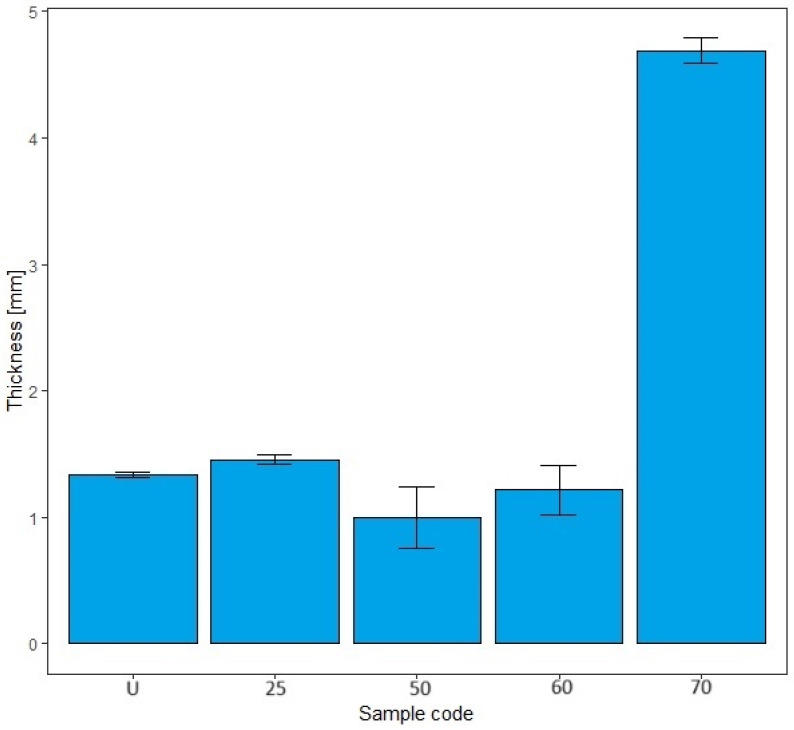
The results of the determination of the thickness of the elastic bandage of the unwashed sample and after 25, 50, 60, and 70 washing cycles in standard detergent.

The results obtained in this study (mass per unit area, thickness) indicate fibrillation of the cotton fibers, which causes the loss of the dense texture of the bandages and thus the rubber thread with an increasing number of washing cycles. As the number of washes increases, the tightness decreases and the cotton fibers fibrillate, causing the rubber threads to fray and deteriorate. The properties of rubber threads are determined by the number of additives added during molding to improve the shortcomings of the rubber thread itself. Rubber threads are protected with cotton yarn in elastic bandages. In this work, it can be assumed that the sheathing of the cotton yarn is separated from the rubber thread during washing and mechanical launching, allowing direct contact with the detergents. Like all polymers, the rubber threads also degrade over time. The degradation of rubber can lead to hardening or softening, depending on the structure of the rubber [43]. Hardening occurs more frequently because free radicals generated by detergents, heat, water, oxygen, etc., form new cross-links, reducing the flexibility of the rubber threads [43]. The degradation of rubber can lead to hardening or softening, depending on the structure of the rubber, with hardening occurring more frequently because free radicals generated by detergents, heat, water, oxygen, etc., form new cross-links and reduce the flexibility of the rubber threads [43]. Although the general mechanism of autoxidation is well understood, the actual steps of chain scission and cross-linking are often unknown. They depend on the composition of the rubber, including the concentration of accelerators, activators, and fillers, as well as the temperature and composition of the atmosphere [43,44,45]. In this work, the properties of the rubber fibers were certainly influenced by the detergents, the washing temperature, the mechanical movement during washing, and the number of washing cycles. The influence of these factors can be seen in the results of the construction properties as well as the tensile and elasticity properties from Section 3.2, Section 3.3 and Section 3.4. The results shown for mass per unit area and thickness are the arithmetic mean of five measurements taken at different points on the elastic bandages after washing and also on the unwashed sample. The error bars have been added to the diagrams in Figure 4, Figure 5, Figure 6 and Figure 7 to indicate the accuracy of a measurement. The bars represent the standard deviation and show how far the true value is from the determined value.

The results of the determination of the mass per unit area of the elastic bandage are shown in Figure 4 and Figure 5. The results of the determination of the mass per unit area of the elastic bandage show that the values of the mass per unit area decrease with increasing number of washing cycles in standard soap and standard detergent.

The reduction in mass per unit area is gradual and the values decrease as follows: U > 25 > 50 > 60 > 70. In Figure 4 and Figure 5, the error bars do not overlap. The results of mass per unit area are highly significant, with a confidence interval of 95%. The values of the mass per unit area after washing in standard soap are lower than in standard detergent for sample marks 25, 50, and 60. For sample mark 70, the value of the mass per unit area after washing with standard soap is higher than with standard detergent. The reason for this is the composition of the detergent because the SDC standard detergent has a more complex chemical composition than standard soap. According to the safety data sheet, the standard detergent used in this work contains a defoamer, soda ash, and sodium disilicate and it is a reaction product of benzenesulfonic acid, 4-C10-13-sec alkyl derivatives and benzenesulfonic acid, 4-methyl, and sodium hydroxide. If we take into account the composition of the standard detergent, the processing time (15 min), the processing temperature (40 °C), and the mechanical agitation of the samples in AUTOWASH, we can conclude that there was a loss of cotton fibers from the yarn of the elastic bandage in the detergent bath. The mass per unit area decreased from 251 g/m^2^ to 185 g/m^2^ when washing with standard detergent. When washing with standard soap, it decreased from 251 g/m^2^ to 203 g/m^2^. The use of non-corrodible stainless steel balls, which have an abrasive effect in the washing tests to better simulate washing under real conditions, had no effect on the thickness (no drastic reduction) and had no effect on the mass per unit area. The above explanations can confirm the assumption that the decrease in mass per unit area is due to the release of excess fibers and fibers that have not been sufficiently spun into the yarn of the elastic bandage. This is confirmed by the results of the thickness determination, where the fiber loss was not excessive and did not significantly affect the results of the thickness determination (Figure 6 and Figure 7). The thickness value increased significantly after 70 washes in standard detergent, which is probably a result of the numerous washes that led to the formation of loops.

The thickness values show the following behavior U > 25 < 50 > 60 < 70 (standard soap) and U < 25 > 50 < 60 > 70 (standard detergent) in Figure 6 and Figure 7. In Figure 6, the error bars overlap by more than 25%; our thickness value results do not appear to be significant. This could be because there is no significant difference between the samples. In Figure 7, the error bars for all samples except sample code 70 overlap by more than 25%. Our results do not appear to be significant, except for sample code 70. For the samples with codes U, 25, 50, and 60, this could be because there is no significant difference between the thicknesses of the samples. For sample code 70, the error bars do not overlap at all; our results for thickness are significant, with a confidence interval of 95%. For thickness, the results after washing in the standard soap solution are higher for samples 50 and 70. The thickness values are higher for the standard detergent after 25 and 60 washes. Based on all of the above and taking into account the specificity of the sample and its users, the number of wash cycles that would have to be determined as optimal would be 60 cycles with the standard detergent. It is assumed that a significant increase in the thickness of the sample after 70 consecutive wash cycles in standard detergent could be due to loop formation during mechanical agitation. The loops led to an increase in the thickness value, but not in the mass per unit area. As already mentioned, the mass per unit area results depend on the fibers that are more or less spun in the elastic bandage. They remain in the detergent bath during mechanical agitation.

### 3.2. Results of the Determination of Maximum Force and Elongation

The tensile strength of the elastic bandages was tested. The main component of the elastic bandages is a rubber thread, whose strength is good but depends on the degree of vulcanization and the crystallinity of the structure. Therefore, it was necessary to determine the maximum force, elongation, and the results of the *t*-test, as shown in Table 2 and Table 3. A statistical *t*-test was performed to determine whether the wash cycles had an effect on the tensile properties of the elastic bandages. It was hypothesized that there is no difference in the mean value between the variables of the unwashed and washed samples. The alternative hypothesis was that there was a difference in the mean value between the variables of the unwashed and washed samples. The unwashed sample (U) was statistically analyzed in relation to the 25 washed samples (25), as was the unwashed sample (U) in relation to the 50, 60, and 70 washed samples. The results of the maximum force and elongation were subjected to the *t*-test.

The hypothesis tested was the null hypothesis, which states that there is no significant difference between the means of the unwashed and washed samples. We performed a *t*-test to determine whether the differences between the elongation values of the samples after different wash cycles (25, 50, 60, and 70) and the unwashed sample (U) were statistically significant.

For the samples tested in standard soap, the hypothesis for elongation was rejected for sample codes 25, 50, and 60 as the *p*-values (0.0215, 0.019, and 0.0203, respectively) were below the significance level (α = 0.05). This shows that the elongation of these washed samples differs significantly from that of the unwashed sample.

The rejection of the hypothesis for these samples indicates that the washing cycles (especially up to 60) significantly affected the elongation properties, which is probably due to degradation or changes in fiber structure. For the sample with code 70, the hypothesis was retained as the *p*-value (0.0524) was slightly above 0.05. This indicates that the elongation of the sample after 70 wash cycles is not significantly different from that of the unwashed sample, possibly due to stabilization or relaxation of the fibers after prolonged washing.

For the samples tested in standard detergent, the hypothesis was accepted for sample codes 60 and 70 as the *p*-values (0.3728 and 0.4148, respectively) exceeded the significance level (α = 0.05). This means that the washing process did not significantly change the elongation compared to the unwashed sample, indicating greater stability or resistance to changes in elongation properties under the given conditions.

However, for the samples with codes 25 and 50, the hypothesis was rejected, as the *p*-value (0 and 0.0244) was less than 0.05, indicating that this particular wash caused a significant change in elongation. This difference could be due to a critical threshold effect, where the elastic components experience a temporary degradation, after which the strain stabilizes in subsequent cycles. The variability in the acceptance and rejection of hypotheses can be attributed in particular to the specific responses of polymer (material) to repeated washing:

- Degradation of synthetic polyisoprene (rubber), which can weaken with a higher number of wash cycles;
- Mechanical stress and fiber relaxation, which lead to temporary stabilization after a critical number of cycles;
- Micro-cracks and permanent stretching of the cotton fibers, which can affect the overall elongation;
- Differences between the interaction of soap and detergent with the fiber structure, with the soap softening the cotton while the detergent promotes cohesion of the fiber.

Table 2 and Table 3 show how standard soap and standard detergents affect the elongation of elastic bandages. This could be because synthetic polyisoprene (rubber) gradually weakens, i.e., it can degrade through a higher number of wash cycles with mechanical agitation. In addition, the elongation results could be affected by micro-cracks in the fibers that form after a higher number of cycles and reduce the elastic tension, resulting in greater extensibility. The elastic bandage may also have relaxed because it has been washed and stretched several times. Normal soap can also soften the cotton fibers, making it easier to stretch the elastic segments together with the cotton. Cotton fibers can also stretch permanently over time and lose their original strength, which also contributes to greater elasticity.

The process of fiber restructuring and the removal of surface impurities, which leads to greater cohesion and mechanical stability, can explain the increase in the maximum force of elastic bandages (standard soap and detergent). After 60 washing cycles, the elastic bandage shows a stabilization of the properties, which indicates the achievement of permanent resistance. During washing, especially in the first cycles (up to 25 times), the fibers can become denser and surface impurities or residues from the production process can be removed. This can lead to increased fiber cohesion, which increases the tensile strength of the material and results in a higher breaking force. Standard soap (SDCE type 1) can remove greasy residues and impurities and thus improve the bond between the fibers. Standard detergent substances (SDCE type 2) can reduce the friction between the fibers so that they bond better after drying.

### 3.3. Results of the Determination of the Elasticity of Elastic Bandages

The results of the determination of the elasticity of elastic bandages are shown in Table 4, Table 5 and Table 6. As five cycles with a load of 74.0 N were selected for each sample in accordance with the manual and the ISO mentioned, they are listed in Table 4. In each cycle shown, four phases are observed: the loading phase, the pause during loading, the unloading phase, and the pause at the end of the unloading phase. The elastic bandages with a proportion of wrapped rubber have pronounced elasticity properties. This can be seen from the results of the cyclic loading and unloading test. From cycle 1 to cycle 5, there is a clear trend from left to right towards increasing hysteresis from sample code U to sample code 70 for standard soap and detergent.

The diagram of samples shows that when a bandage is stretched, it produces higher tensions than when it is relaxed. This behavior of elastic bandages is very important as it affects the compression they exert on the limbs when the dimensions of the limbs change [46]. In the next loading cycle, the initial modulus is lower than that of the first loading cycle and the modulus in the direction of maximum extension is higher than that of the first loading cycle. This could indicate that the tension generated and the pressure exerted on the limb are different when a bandage is repeatedly stretched [46].

All sample codes show the range in which the modulus varies rapidly. This range is important for elastic bandages. The bandage is usually stretched in such a way that it is overstretched in this area when it is applied. Varying the wash cycles and the standard soap or detergent has no visible effect on the shape of the curve. In diagrams, all curves have a similar shape. The value of the extensions (mm) recorded for the load (74 N) is very different, as shown later in Table 5 and Table 6.

The mean values of the measurement of the elasticity properties of elastic bandages, the extension at maximum force (E), elongation (S), permanent deformation (C), recovered elongation (D), and elastic recovery (R) according to ISO 20932-3:2018 are listed in Table 5 and Table 6. Equations (1)–(4) were used to calculate the values. The results of the elasticity properties show that the samples of elastic bandages return to their original size and shape after deformation or not, as shown in the tables.

The results of testing the elastic properties of bandages in standard soap and standard detergent show different changes after a certain number of washes, which is of particular importance for assessing the durability and functionality of elastic bandages in clinical and everyday use. The value of elongation at maximum force (E) decreases with an increasing number of washes in standard soap and in standard detergent up to sample code 50. It is assumed that this trend indicates that the elastic fibers degrade through repeated washing, which leads to a decrease in the elastic resistance of the material. However, in the samples with codes 60 and 70, the elongation value increases again, especially with the standard detergent (70 cycles). It is assumed that this phenomenon is due to a restructuring of the fibers or the loss of the previous stiffness of the elastic segment, which allows for greater elongation. In addition, it is possible that the rubber has relaxed and stabilized after a large number of washes.

The unwashed sample shows the highest elongation value (S), while the sample with code 25 also retains a relatively high value. The values then decrease with the number of wash cycles in the standard soap, while an increase is recorded again in the standard detergent in samples 60 and 70. The initial decrease could be due to damage to the elastic fibers caused by the mechanical stress during washing, while the later increase could be the result of a gradual loosening of the fibers and a reduction in tension allowing for greater elongation.

The permanent deformation values (C) increase with the number of washes, indicating a permanent elongation of the material.

The values of strain recovery (D) decrease with increasing number of washes, with the highest value for the unwashed sample (99%) and the lowest (88%) for the sample with 70 washes (detergent). It is assumed that the decrease in restoring force indicates a loss of elastic fiber memory, while the increase in permanent deformation indicates permanent changes in the fabric structure after repeated mechanical stress and chemical washing reactions.

The highest value of elastic recovery (180%) in the sample with 50 wash cycles in standard detergent could be a consequence of structural reorganization of the fibers, which allows them to return to their original shape after stretching. This phenomenon could be due to a temporary increase in fiber flexibility after a certain degradation phase when the fibers lose strength and become more pliable. In the sample with 70 wash cycles, the elastic recovery decreases significantly, indicating a loss of elasticity due to long-term chemical and mechanical degradation.

When analyzing samples washed with the phosphate-free detergent (SDCE type 2), a clear increase in maximum force and elastic recovery can be observed in samples with more cycles, while soap causes a gradual decrease in these properties. It is possible that the detergent interacts differently with rubber and cotton fibers than soap, leading to different changes in elastic properties.

The initial decrease in elasticity, followed by stabilization or increase at higher cycle numbers (60 and 70), can be associated with a restructuring of the fibers and permanent changes in the material.

Based on the results in Table 5 and Table 6 (but also the results in Table 2 and Table 3), it can be assumed that the elastic bandages tested fulfill their function as a compression treatment or support bandage in the long term. They allow compression, which ensures fluid transport through the leg, or are used as support, up to a maximum of 60 wash cycles in standard detergent without significant physical and mechanical changes in properties.

### 3.4. Results of the Analysis of the 3D-Scanned Point Cloud of Legs and Approximation of Compression Bandages

Based on the 3D scan of the human body and the extracted cross-sections of the 3D point cloud, the areas and volumes listed in Table 7 were measured. The cross-sections were defined at a distance of 5 cm, which corresponds to half the width of the compression bandage tested. The measured values are given in mm for the circumferences and mm^2^. The circularity was calculated for all defined cross-sections. The calculation of the approximate compression was based on an adapted Laplace’s law Equation (6), which is used to calculate the compression of cylindrical shapes. Numerous studies used models of human body parts as a basis for shapes, such as the leg, arm, or torso, where it is not possible to clearly determine the radius of the segment, since in a real environment only the circumference of the human body segment can be measured. A circle is the basis for a cylindrical shape, which forms the basis for calculating the compression using Laplace’s law. Starting from a 3D scanned body part, a point cloud of the human body is created. The point cloud of the human body can be used to measure the segment and analyze the shape of body parts, making it possible to perform various numerical analyses. For the purpose of this study, planes were defined that intersect the point cloud at specific positions (Figure 2). The intersection of the plane with the point cloud results in groups of points of the body part on which the circumference and surface area can be determined. This data can be used to determine the circularity of the examined segment, which is not possible with conventional measurements. Previous studies have relied only on the external shape of the human body, and a similar case exists when using a model such as the leg. Human body models, which are often used in research and compression calculations, are created based on average anthropometric measurements and are very often not authentic.

In the approximate calculation of the compression, the number of layers of the bandage was determined and the actual width of the bandage was used. This test was carried out to determine the usefulness of the bandage after a large number of washes by determining the compression according to the force, in this case, 74 N, at which the bandage did not lose its properties after 60 washes. In view of the fact that the radius is a variable quantity in the approximate calculation of compression according to Laplace’s law under the effect of the same force, it is important to determine its value as accurately as possible. Therefore, in this study, the circularity was determined according to expression (5) for the observed segments of the respective cross-sections of the point cloud with transverse planes. If the circularity value is approximately 1, we can speak of a shape close to a circle, while the radius values were calculated based on the circumference of the approximate value of the radius based on the area. The circularity of the shape was calculated for each cross-section of the body at a distance of 5 cm, which gives relevant values (Table 7).

The approximate value of the compression of the bandage was calculated for all cross-sections, assuming the value of the stretching force of the compression bandage around the leg to be 74 N. The value of 74 N was determined using the mass per unit area (251 g m^−2^). The stated force (74 N) corresponds to the ISO 20932-3:2000 standard, which was used for testing the elongation, permanent deformation, restored deformation, and elastic recovery of samples washed in standard detergent and soap. It was shown that the specified properties remained within the limits in which the compression bandage could fulfill the compression function after 60 washes.

**Figure 8:**
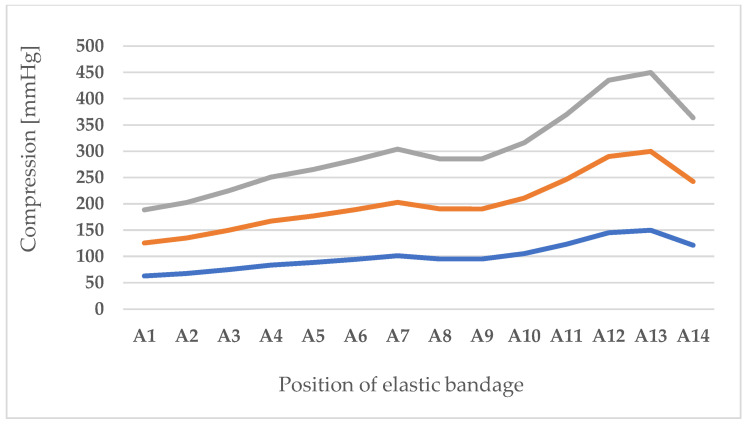
Compression results expressed in mmHg for one layer of bandage (blue), two layers (orange) and three layers (grey).

Table 7 shows the compression results for the circularity value and Figure 8 shows the compression results expressed in mmHg so that they can be compared and commented on with the compression values recommended by medical professionals. The results in Table 7 show that the lowest compression for the largest leg circumference is 8379.8 Pa, which corresponds to 62.8 mmHg. As you decrease the radius for each body cross-section, the compression increases and reaches the highest value of 19,335.37 Pa (145.03 mmHg). The specified compression range refers to one layer of the bandage when a force of 74 N is applied during wrapping. The approximate compression values calculated according to the adjusted Laplace’s law increase linearly with the amount of layers of the bandage used. This means that the bandage can be used for the highest compression prescribed by the standards even after prolonged use. As there are a large number of studies comparing the approximate values determined according to the adapted Laplace’s law with actual measurements, the circularity was calculated for each body cross-section. In this way, it can be determined that the individual segments of the leg can be considered cylindrical shapes for the approximate calculation of the compression of the bending in the first layer by applying Laplace’s law.

The shape of the body influences the compression value and, for this reason, it was very important to analyze and calculate the circularity. The circularity was calculated based on the circumference and area and Equation (5). The results presented in Table 7 show that the circularity values for the thigh and lower leg area are close to 1, which means that the shape of these segments is close to a perfect circle. For the knee area (position A6) and the ankle area (positions A13 and A14), the circularity value deviates from 1. This situation is not entirely commonplace, as human bodies and shapes are very different. Often the knee and ankle areas are also approximately circular due to swelling, which is one of the reasons for using a compression bandage for these areas. Based on the adjusted Laplace’s law, the compression was calculated for each cross-sectional circumference of the leg. From the results shown, the lowest compression occurs at the largest leg circumference (position A1) and is 8379.8 Pa, which corresponds to 62.8 mmHg. As the radius decreases, the compression increases and reaches its highest value at position A13, which could be considered irrelevant for these considerations, as the cross-section in the ankle area does not have a circular cross-section, as the circularity value of 0.885291 shows. Therefore, a value should be selected that corresponds better to the compression. This is the case in the area of position A12, where the calculated compression is 19,335.37 Pa (145.03 mmHg).

The specified compression range applies to a layer of bandage wrapped with a force of 74 N. The lowest value given is greater than the lower limit, which defines the strongest (extra high) compression, regardless of which national standard it refers to. Each additional layer of the bandage provides greater compression. The approximate value of the bandage compression at a force of 74 N was calculated for several layers, as the bandage is not applied in one layer, but always with overlapping layers. The approximate values for compression calculated according to Laplace’s law are increased as often as the number of bandage layers used. In order to achieve results as similar as possible to the actual application, the thickness of the bandage should also be taken into account when calculating the compression values in the second and each subsequent layer. In this work, the influence of the bandage thickness on the compression was not investigated, as the focus is on the approximation of compression and the physical and mechanical properties of polymer materials for bandages. The approximation of the compression of a single, double, or sometimes multi-layer wrapped bandage according to the input parameters shows that the tested bandage can be used for the highest compression even after prolonged use and washing (25, 50, and 60 wash cycles). This is also supported by the results obtained, i.e., the differences in compression measurements per area (A1–A14). Finally, it can be seen that the breaking force of the elastic bandage is much greater than the force used for the approximation. This indicates that although the bandage loses its elastic properties after washing, it can still be used as an effective compression bandage.

### 3.5. Results of the Morphological Characteristics

The morphology of the elastic bandages as determined by Dino-Lite analysis is shown in Table 8 to illustrate the morphological differences between the unwashed and washed samples.

Table 8 clearly shows the method used to produce the elastic bandages and the effects of standard soap and standard detergent on the bandages at different numbers of washes (25, 50, 60, and 70). The figures in Table 8 show that the washing process significantly influences the structure of the elastic bandages, with the standard soap causing more tufting and giving the impression of a softer structure, while the standard detergent contributes to a clearer appearance of the connection points and a more compact structure. The rubber material, i.e., the rubber threads, does not show any visually perceptible deterioration, damage, or anything that visually indicates wear in any of the images. In samples with a lower number of washes (25), the bonding points are clearly visible and almost regular, while in samples with 50 or more washes, they take on an increasingly irregular shape. This is the result of repeated washing with a mechanical start and steel balls. The surface of the samples appears shaggier in samples washed with standard soap, especially after 60 washes, while the detergent produces a uniform and smoother surface.

## 4. Conclusions

This study aimed to examine the impact of washing on the physical and mechanical properties of elastic bandages, focusing on the number of washes after which the bandage retains satisfactory properties. Samples were washed in the AUTOWASH II machine with standard detergents according to ISO 105-C10.

A *t*-test compared elongation values after different wash cycles (25, 50, 60, and 70) with unwashed samples. For soap-washed samples, significant changes in elongation were observed after 25, 50, and 60 cycles (*p* < 0.05), likely due to fiber degradation, while cycle 70 showed stabilization (*p* = 0.0524). For detergent-washed samples, cycles 60 and 70 showed no significant change (*p* > 0.05), while cycles 25 and 50 indicated temporary degradation. Differences in elongation were influenced by fiber degradation, relaxation, micro-cracks, and varying interactions with soap and detergent.

Mechanical properties stabilized after 60 wash cycles, possibly due to fiber restructuring and impurity removal. A decrease in mass per unit area and thickness was noted with more wash cycles. Elongation at maximum force decreased up to 50 cycles but increased after 60 and 70, especially with detergent, suggesting fiber stabilization. Elastic recovery (R) peaked after 50 cycles with detergent, while permanent deformation (C) increased, indicating reduced elastic memory. Up to 60 cycles with detergent preserved acceptable elastic properties.

The other focus of this work is the calculation of bandage compression, for which an adapted Laplace’s law was used. An approximate bandage compression was calculated. The results show that the maximum medical compression can be achieved in one layer of the bandage when a force of 74 N is applied. The bandage overlaps during application and the compression of the tested bandage can reach higher values. The calculated compression is an approximation as there are a number of subjective parameters that affect the required compression of a body part, such as the body shape, the tissue structure, the user’s needs, the friction between the skin surface and the bandage or bandage layers, and the user’s or medical staff’s experience in using a compression bandage. A large number of the parameters listed cannot be clearly determined from the user’s point of view and the calculated compression values are significantly higher than the standard values. It can therefore be concluded that the tested bandage is effective at lower forces and after a greater number of washes and applications.

## Figures and Tables

**Table 1:** Sample codes.

Sample Code	U	25	50	60	70
Treatment	Unwashed	25 washing cycles	50 washing cycles	60 washing cycles	70 washing cycles

**Table 2:** The strength of the tested elastic bandages in the standard soap.

Property		F [N]					ɛ [%]				
Sample Code		U	25	50	60	70	U	25	50	60	70
n	1	299.8	368.7	237.1	361.6	250.7	179.3	207.6	233.7	216.3	163.8
	2	382.9	238.7	245.9	143.3	351.9	173.1	178.5	209.1	175.2	272.7
	3	334.4	329.2	257.7	287.7	324.3	179.7	222.9	192.3	209.1	255.3
	4	250.7	343.8	255.9	281.7	278.1	153.7	226.8	273.6	206.4	196.6
	5	325.0	417.7	291.7	326.0	320.4	157.9	183.0	201.3	182.1	236.1
	X	318.6	339.6	257.7	280.1	305.1	168.7	203.8	222.0	197.8	224.9
	σ	43.34	58.74	18.56	74.19	36.00	10.90	19.90	29.25	16.13	39.67
	CV [%]	13.60	17.30	7.20	26.49	11.80	6.46	9.76	13.18	8.15	17.64
*t*-test	t-stat	/	−0.577	2.5837	0.8962	0.4785	/	−3.0861	−3.4128	−2.9871	−2.7301
	t-crit	/	2.3646	2.57054	2.4469	2.36462	/	2.4469	2.57054	2.36462	2.7763
	df	/	7	5	6	7	/	6	5	7	4
	*p*-value	/	0.582	0.0492	0.4047	0.6469	/	0.0215	0.019	0.0203	0.0524
	Decision	/	+	−	+	+	/	−	−	−	+

F [N]—breaking force; ɛ [%]—elongation at break according to ISO 13934-1:2008; and U—unwashed sample and properties of elastic bandages after 25, 50, 60, and 70 washing cycles. *t*-test = Student’s *t*-test; df = degrees of freedom for the *t*-test; t-stat = test statistic t; and *p* = value for a two-tailed *t*-test, t—critical = two-tailed t-critical test, decision = acceptance (+) or rejection (−) of the hypothesis: there is no difference in the mean value between the variables of the unwashed and washed samples.

**Table 3:** The strength of the tested elastic bandages in the standard detergent.

Property		F [N]					ɛ [%]				
Sample Code		U	25	50	60	70	U	25	50	60	70
n	1	299.8	428.2	330.2	331.7	195.2	179.3	244.2	317.9	177.6	106.5
	2	382.9	414.4	355.9	418.5	360.4	173.1	286.5	308.1	219.0	164.8
	3	334.4	370.9	323.7	334.9	369.7	179.7	286.1	262.5	187.9	173.1
	4	250.7	312.3	369.7	391.2	266.0	153.7	299.4	233.0	182.3	177.9
	5	325.0	365.5	286.8	200.9	292.4	157.9	283.9	179.4	142.4	159.3
	X	318.6	378.3	333.3	335.4	296.7	168.7	280.0	260.2	181.8	156.3
	σ	43.34	40.91	28.63	74.99	64.26	10.90	18.72	50.79	24.46	25.73
	CV [%]	13.60	10.81	8.59	22.36	21.66	6.46	6.69	19.52	13.45	16.46
*t*-test	t-stat	/	−2.0036	−0.566	−0.3898	0.563	/	−10.2724	−3.5206	−0.9784	0.8889
	t-crit	/	2.36462	2.4469	2.4469	2.36462	/	2.4469	2.7763	2.57054	2.57054
	df	/	7	6	6	7	/	6	4	5	5
	*p*	/	0.0852	0.5919	0.7102	0.591	/	0	0.0244	0.3728	0.4148
	Decision	/	+	+	+	+	/	−	−	+	+

F [N]—breaking force; ɛ [%]—elongation at break according to ISO 13934-1:2008; and U—unwashed sample and properties of elastic bandages after 25, 50, 60, and 70 washing cycles. *t*-test = Student’s *t*-test; df = degrees of freedom for the *t*-test; t-stat = test statistic t; and *p* = value for a two-tailed *t*-test, t—critical = two-tailed t-critical test, decision = acceptance (+) or rejection (−) of the hypothesis: there is no difference in the mean value between the variables of unwashed and washed samples.

**Table 4:** The results of the cyclic loading and unloading test.

	Treatment	Unwashed	Standard Soap				Standard Detergent			
	Sample Code	U	25	50	60	70	25	50	60	70
Cycles	Phase	E [mm]	E [mm]	E [mm]	E [mm]	E [mm]	E [mm]	E [mm]	E [mm]	E [mm]
1st	Loading	200.8	204.5	207.7	202.0	172.7	255.4	116.8	107.8	170.6
	Unloading	/	/	/	/	/	/	/	/	/
2nd	Loading	301.9	205.9	208.8	203.8	174.1	256.3	117.3	209.2	172.2
	Unloading	16.7	21.1	21.2	203.6	26.9	22.5	10.9	103.6	19.4
3rd	Loading	302.9	277.2	210.4	205.0	175.5	261.2	152.9	210.2	305.8
	Unloading	17.8	22.2	21.2	204.2	28.7	22.9	11.6	112.2	20.0
4th	Loading	302.5	206.8	209.9	204.8	174.8	257.6	118.1	209.8	173.0
	Unloading	18.2	22.6	21.8	204.7	29.1	24.3	12.2	164.8	20.2
5th	Loading	302.7	206.9	210.0	205.3	175.3	257.6	118.1	210.1	173.3
	Unloading	18.5	23.2	22.4	205.2	29.3	23.9	11.9	114.2	20.9

E is the extension at maximum force; in accordance with the manual and ISO 20932-3:2018, the load was 74.0 N and the recovery time was 1 min. The extension and retraction speed of the sample was set to 100%/min. The gauge length was (100 ± 1) mm. Each cycle is divided into four phases: the loading phase, the pause under load, the unloading phase, and the pause at the end of the unloading phase.

**Table 5:** The results of the elasticity properties of elastic bandages in standard soap.

Sample Code	E [mm]	S [%]	C [%]	D [%]	R [%]
U	302.9	202.7	1	99	48.9
25	277.2	177.4	4	96	54.1
50	210.4	110	7	93	84.6
60	205.0	105.3	10	90	85.5
70	175.5	75.3	10	90	119.5

E is the extension at maximum force; S is elongation; C is the permanent deformation; D is the recovered elongation; and R is the elastic recovery.

**Table 6:** The results of the elasticity properties of elastic bandages in standard detergent.

Sample Code	E [mm]	S [%]	C [%]	D [%]	R [%]
U	302.9	202.7	1	99	48.6
25	261.2	161.5	3	97	60.1
50	152.9	52.5	5	95	180.9
60	210.2	110.1	9	91	82.7
70	305.8	205.7	12	88	42.8

E is the extension at maximum force; S is elongation; C is the permanent deformation; D is the recovered elongation; and R is the elastic recovery.

**Table 7:** The results of the perimeter, area, circularity, and approximate compression values for one-, two-, and three-layer bandages for 3D-scanned leg sections.

Position	Girth [mm]	Area [mm^2^]	Circularity	Compression [Pa] n = 1	Compression [Pa] n = 2	Compression [Pa] n = 3
A1	554.853	24,305.22	0.992096	8379.8	16,759.6	25,139.4
A2	516.547	21,054.66	0.991604	9001.228	18,002.46	27,003.68
A3	465.392	17,100.94	0.992184	9990.625	19,981.25	29,971.88
A4	417.343	13,687.74	0.987541	11,140.85	22,281.71	33,422.56
A5	394.242	11,893.01	0.961559	11,793.66	23,587.33	35,380.99
A6	369.099	10,211.21	0.941893	12,597.05	25,194.09	37,791.14
A7	344.176	9329.952	0.989756	13,509.24	27,018.49	40,527.73
A8	366.373	10,498.71	0.982877	12,690.77	25,381.55	38,072.32
A9	366.612	10,495.31	0.981277	12,682.5	25,365	38,047.5
A10	330.94	8587.916	0.98537	14,049.55	28,099.09	42,148.64
A11	283.033	6297.931	0.987947	16,427.61	32,855.23	49,282.84
A12	240.469	4456.471	0.968462	19,335.37	38,670.74	58,006.11
A13	232.634	3812.612	0.885291	19,986.58	39,973.15	59,959.73
A14	287.647	5078.007	0.771229	16,164.11	32,328.22	48,492.32

**Table 8:** Morphological characteristics.

Code	Morphological Characteristics	
	Standard Soap	Standard Detergent
25	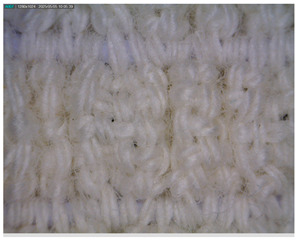	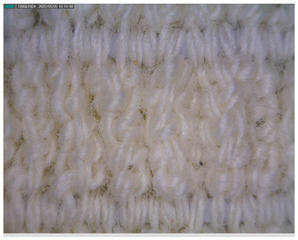
50	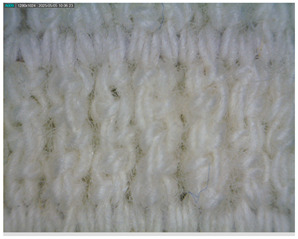	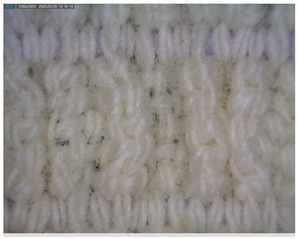
60	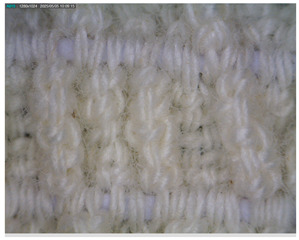	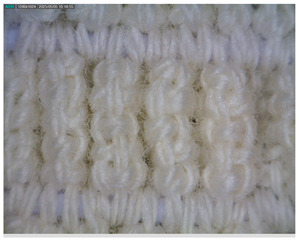
70	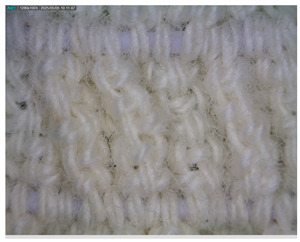	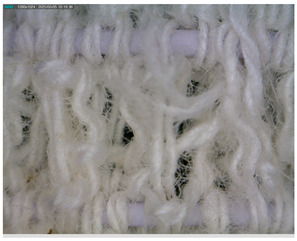
U	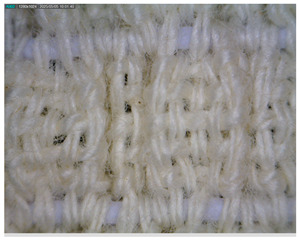	

## Data Availability

The original contributions presented in this study are included in the article; further inquiries can be directed to the corresponding author.
